# Sex and Isolated Anthropometric Measures Do Not Explain Individual Differences in Responsiveness to Advanced Footwear Technology in Highly Trained Runners

**DOI:** 10.1111/sms.70234

**Published:** 2026-02-22

**Authors:** Ieva Seglina, Kalle Torniainen, Louise Kvarforth, Stefan Hallström, Anton Arndt

**Affiliations:** ^1^ The Swedish School of Sport and Health Sciences (GIH) Stockholm Sweden; ^2^ KTH Royal Institute of Technology Stockholm Sweden; ^3^ Karolinska Institute Stockholm Sweden

**Keywords:** advanced footwear technology, anthropometry, long‐distance runners, running economy, sex differences

## Abstract

Advanced footwear technology (AFT) has improved running economy in distance runners, yet individual responsiveness varies widely. Sex differences and anthropometric characteristics have been proposed as potential factors, but evidence remains limited. This study investigated whether sex‐based differences exist in the response to running in an AFT shoe compared to a non‐AFT control shoe. The response was measured by means of the change in energy cost (EC), ΔEC, and it was assessed whether isolated anthropometric characteristics could predict ΔEC in highly trained long‐distance runners. Fifteen female and fifteen male runners completed treadmill running economy tests at 60%, 70%, and 80% of their VO_2peak_ speed in AFT and non‐AFT shoes. Anthropometric measures investigated were height, body mass, foot length, femur and tibia length, and Achilles tendon length. Females showed significantly lower absolute EC than males across all speeds and shoe conditions (*p* < 0.05), but ΔEC did not differ between sexes (*p* = 0.5). Average individual ΔEC ranged from 1.1% to 6.4% in females and 0.2% to 8.7% in males. No anthropometric variable could significantly predict ΔEC in sex‐stratified analyses. Highly trained runners exhibit large inter‐individual variability in responsiveness to AFT shoes, but neither sex nor isolated anthropometric traits explained these differences in this study. These findings highlight the complexity of AFT individualization and suggest that personalization should not be based solely on sex or the anthropometric characteristics investigated in this study.

## 1 Introduction

Since the introduction of long‐distance running “super‐shoes” in the mid‐2010s, both elite athletes and recreational runners have adopted shoes with advanced footwear technology (AFT). These shoes feature a lightweight construction with a high‐energy return midsole foam, a longitudinal rigid element in the midsole, and a pronounced rocker profile in the sole [1]. The benefit of wearing AFT shoes is commonly measured through running economy (RE), defined as the rate of oxygen uptake required to run at a specific speed (mL·kg^−1^·min^−1^), or through energetic cost (EC), which accounts for substrate utilization (W·kg^−1^), with changes in EC (ΔEC) between running in non‐AFT and AFT shoes serving as key indicator of shoe responsiveness.

The first study measuring EC in the new Nike prototype shoe with AFT properties showed that, on average, the shoe lowered EC by 4% in high‐caliber athletes compared to a conventional marathon shoe without AFT [2]. This claim has later been confirmed across different AFT shoe models, runner experience levels, and running speeds, with reported average improvements in RE ranging from 2.8% to 4.4%, consistent with earlier EC findings [3, 4, 5, 6, 7]. Since the launch of AFT shoes, world records in middle‐ and long‐distance running have improved greatly. Notably, female runners appear to achieve greater reductions in race times in AFT shoes than their male counterparts, especially in longer distances [8, 9].

Despite the consistent average improvements in RE and EC with AFT shoes, individual responses vary widely. For example, Riedl et al. [10] showed that male amateur triathletes had a −4.5% to 5.8% improvement in EC wearing a specific AFT model, while Knopp et al. [11] observed even greater inter‐individual variability in world‐class Kenyan runners, one AFT model showed changes in RE ranging from ~−5% to 11.4%, while a different AFT model showed changes ranging from −11.3% to ~5%.

The mechanisms underlying runners' responses to AFT shoes can be conceptualized via two complementary pathways: direct (e.g., plate stiffness [12], sole rocker profile [13], and midsole energy return [14]) and mediated biomechanical effects, as proposed by Connick and Lichtwark [15]. While the framework proposed by Connick and Lichtwark recognizes inter‐individual variability, it does not incorporate how individual athlete characteristics (e.g., physiology, anthropometry, movement patterns) moderate the runner‐shoe interaction [8, 9].

Running economy is influenced by a combination of biomechanical, physiological, and anthropometric factors [16]. Among anthropometric traits, height, body mass, and limb dimensions have been associated with RE [16]. For example, taller runners and those with longer legs tend to exhibit better RE and performance [17, 18, 19]. In habitual middle‐distance runners, an inverse relationship exists between body mass and RE, where greater body mass is associated with better RE [17, 20]. Additionally, longer Achilles tendons (AT) have been associated with lower EC [21].

Although these traits are linked to RE in general, their role in modifying changes in RE, specifically due to footwear, remains unclear. Factors such as body mass, shoe size, and leg length have been proposed as potential contributors to improved performance for the world's best runners in AFT shoes [8, 9]. Although RE and performance are two distinct measures, there is an established association between the two [16, 22, 23, 24]. Given this relationship, Riedl et al. [10] found that lighter runners showed greater improvements in RE in one of the four tested AFT shoe models. On the other hand, Van Hooren et al. [25] found no relationship between improvement in EC and body mas or foot length. However, despite their relatively large sample of runners (*n* = 41), they did not stratify analyses by sex, which may have prevented the identification of sex‐specific differences.

Given the variability in individual responses to AFT shoes and the unclear role of anthropometric traits in moderating these effects, further investigation is warranted. Identifying whether characteristics such as body mass, height, leg length, foot size, or AT length influence the energetic cost benefit of running in AFT shoes could help support the development of personalized footwear recommendations, thereby maximizing EC improvements for runners [15]. In line with recent recommendations to disaggregate data by sex [8, 9], this study analyzed female and male runners separately to better understand potential sex‐based differences in their responses to AFT shoes.

Accordingly, this study aimed to explore whether sex‐based differences exist in the change in EC of running induced by AFT shoes, and to examine whether anthropometric characteristics are associated with these changes in highly trained long‐distance runners. We hypothesized that (1) females would exhibit a greater ΔEC benefit than males, and (2) specific anthropometric traits would influence ΔEC, with these associations differing between sexes.

## 2 Materials and Methods

### 2.1 Participants

15 female (age: 32.4 ± 4.7 years; height: 167.6 ± 4.7 cm; body mass: 57.0 ± 4.9 kg) and 15 male (age: 31.3 ± 6.7 years; height: 179.4 ± 7.2 cm; body mass: 67.2 ± 7.6 kg) highly trained/national level [26] long‐distance runners volunteered to participate in the study. Twenty‐eight participants reported regular use of AFT shoes during training and/or competition, with two not specifying the shoe model. The inclusion criteria were: between 18 and 45 years old; free from injury over the previous 3 months, and a recent 10 km time under 39:00 min for females (36:59 min ± 1:40 min) and 34:00 min for males (30:57 min ± 1:42 min). The sample size was determined by the availability of eligible participants and logistical feasibility; no a priori sample size calculation was performed. All participants were informed of the risks and benefits of the study and gave written consent to participate. The study was approved by the Swedish Ethical Review Authority (Dnr 2022‐05850‐02).

### 2.2 Experimental Design

The experimental protocol consisted of 3 days of visits within a week, separated by at least 24 h. At the first visit, anthropometric parameters and lactate threshold were measured, and aerobic capacity (VO_2peak_) was tested. At the second visit, the participants completed running economy tests, while at the third visit, their AT length and maximal strength were measured. Maximal strength data, however, are not included in the present analysis, as they fall outside the scope of this study.

Participants were required to follow a standardized protocol before all three visits. It included a recommended sleep of 8 h, no intake of nitrate‐rich foods or caffeine, no training 24 h before the testing, and no intake of food 2 h before the testing.

### 2.3 Anthropometric Measurements

Participants' height and mass were measured using a manual height meter and a digital scale, to the nearest 0.1 cm and 0.1 kg, respectively. The foot length was measured by having the participant stand on a piece of paper with the heel against the wall, and the length was manually measured from heel to toe.

Supine Dual‐energy X‐ray Absorptiometry (DXA) (Lunar Prodigy Advance; GE Healthcare, Little Chalfont, United Kingdom) examination was performed to measure the bone length of the tibia and femur. Before the examination, the device was calibrated according to the manufacturer's instructions.

**FIGURE 1:**
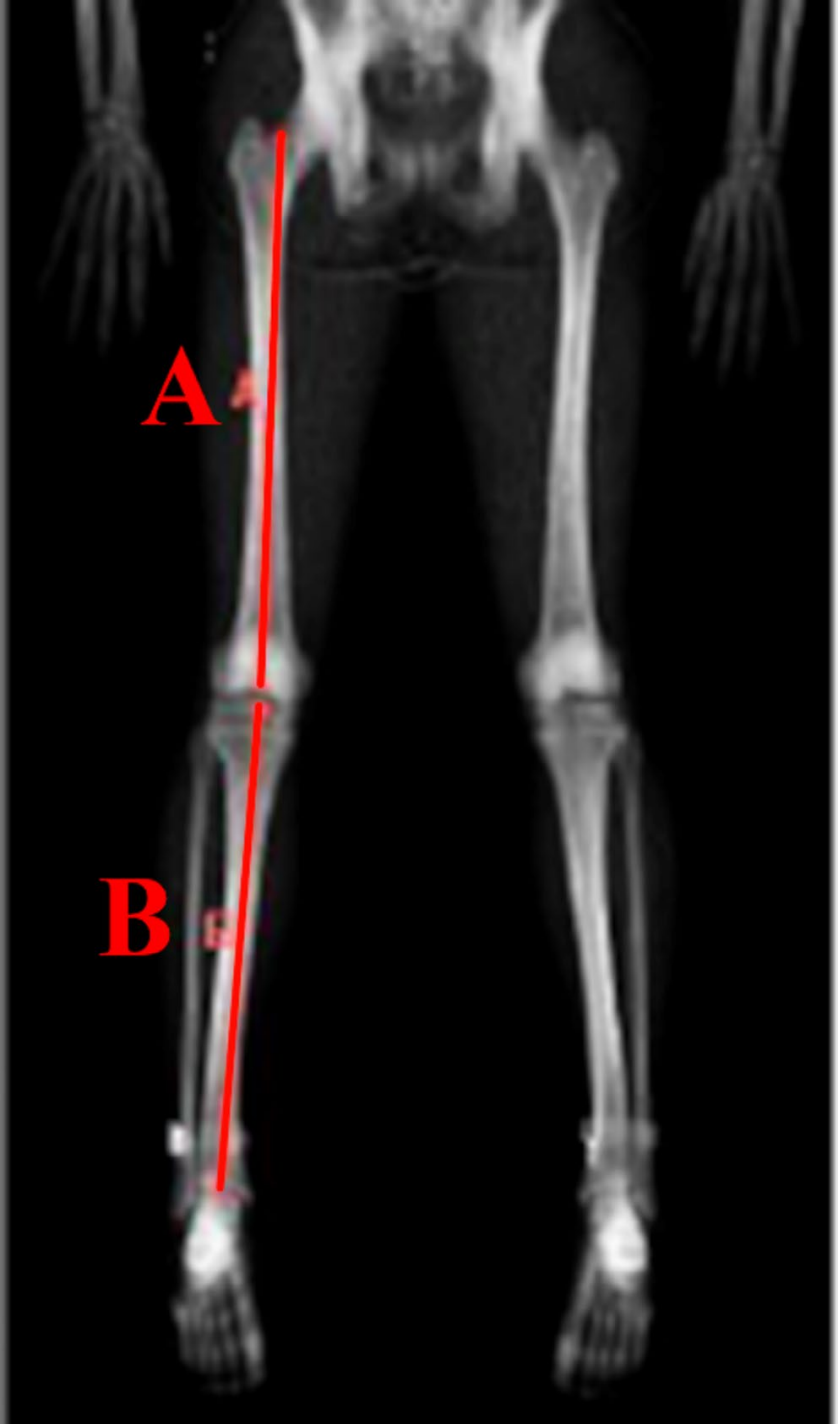
Example measurement of femoral length (A) and tibial length (B).

Femoral length was measured as the linear distance from the base of the femoral neck to the midpoint between the medial and lateral femoral condyles. Tibial length was measured as the linear distance from the intercondylar eminence to the midpoint of the tibial plafond (Figure 1). All measurements were obtained from the right limb of the participant.

**FIGURE 2:**
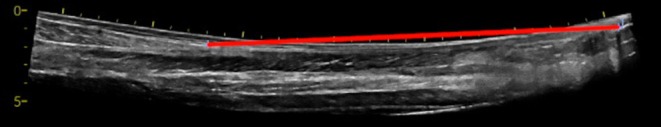
Example measurement of AT length. The red line shows the measured tendon length.

The length of the AT was measured using ultrasound (Vivid iq, General Electric Company, Horten, Norway) when the participants were in a supine position and relaxed with their ankle positioned at the end of the bed. The test leader used their knee/thigh to place the participant's ankle in 90° of passive dorsiflexion to standardize the procedure and avoid ankle dorsiflexion/plantarflexion and pronation/supination. The length of the AT was measured from the most distal part of the calcaneal insertion of the AT to the medial gastrocnemius myotendinous junction (MTJ), as detected sonographically and recorded through the panorama function (Figure 2), which enabled the calculation of the AT using the software's instrument for measuring length/distance [27].

### 2.4 Lactate Threshold and Aerobic Capacity

All running tests were performed on a treadmill (Rodby RL2700E, Vänge, Sweden) set at a 1% incline, in a climate‐controlled room set to 19°C and 40% humidity. The participants completed lactate threshold and aerobic capacity tests in their own, freely chosen running shoes, with specific shoe models not recorded.

Participants first performed a 10‐min warmup on the treadmill at a self‐selected speed. The lactate threshold test followed a ramp protocol where the initial speed was set at 60% of their season best speed for a 10 km race, increasing by 10% of their season best speed at each stage. Each stage lasted 4 min, followed by a 1‐min rest, during which a finger‐prick blood sample was taken and analyzed with an automated analyzer (Biosen 5140, EKF Diagnostics, Barleben, Germany). The test continued until the participant's lactate level exceeded 4 mMol·L^−1^.

Oxygen uptake measurements (VO_2peak_) were completed after the lactate threshold test, with a 7‐min rest in between. Before each athlete's visit, the respiratory gas analyzer Oxycon Pro (Erich Jaeger GmbH, Hoechberg, Germany) was calibrated according to the manufacturer's instructions. The VO_2peak_ test followed a stepwise protocol, increasing speed until voluntary exhaustion or when an oxygen uptake plateau was observed. The initial speed was set at 80% of their 10 km race season best, with 5% increments of initial speed every minute, until the participant reached exhaustion or an oxygen uptake plateau was observed.

### 2.5 Energetic Cost

As part of a broader footwear testing protocol, participants completed EC testing across four shoe models during the second visit (three AFT and one non‐AFT). The shoe order was randomized with a Latin Square design. Participants performed a 10‐min warm‐up routine on the treadmill in their own running shoes at a self‐selected speed. After the warm‐up, participants completed 4 min of running at each intensity of 60%, 70%, and 80% of their VO_2peak_ speed with four different shoes (Table 1). VO_2_ was measured continuously throughout each 4‐min bout, and the mean VO_2_ over the last 2 min was used for analysis. There was a 5‐min rest between the shoe models. If the respiratory exchange ratio (RER) value exceeded 1.0 at any time during the trial, it was excluded from further analysis to ensure that the running intensity did not exceed the anaerobic threshold. After the participants had completed the run with one of the shoes, a finger‐prick blood sample was taken to analyze the lactate levels. The last trial (at 80% of VO_2peak_ speed) was excluded if lactate levels exceeded 4 mMol·L^−1^ [28].

**TABLE 1:** Individual running speeds (km·h^−1^).

	60% of VO_2peak_ speed	70% of VO_2peak_ speed	80% of VO_2peak_ speed
Female	10.2 ± 0.4 (9.4–11.0)	12.0 ± 0.5 (10.9–12.9)	13.7 ± 0.6 (12.5–14.7)
Male	12.0 ± 0.7 (10.7–13.0)	14.0 ± 0.8 (12.5–15.1)	16.0 ± 0.9 (14.3–17.3)

*Note:* Data presented as: mean ± SD (range).

The mean oxygen [mL·kg^−1^·min^−1^] consumption and RER values were determined by averaging the last minute at each speed. Further, the energetic cost of running [W·kg^−1^] was calculated by Brouwer's equation [29]:

$$\begin{array}{c} \mathrm{EC}\;\left[W\cdot {\mathrm{kg}}^{-1}\right]=\\ {\mathrm{VO}}_{2}\times \left(\left(\frac{\mathrm{RER}-0.71}{0.29}\times 21.4\right)+\left(\frac{1-\mathrm{RER}}{0.29}\times 19.4\right)\right)/60 \end{array}$$

Afterwards, the change in energetic cost (ΔEC) between the AFT and non‐AFT shoe (shoe conditions described below) was calculated as follows:

$$\mathrm{\Delta EC}\;\left[\%\right]=\left(1-\frac{\mathrm{AFT}}{\text{non}‐\mathrm{AFT}}\;\right)\times 100$$

### 2.6 Shoe Conditions

**FIGURE 3:**
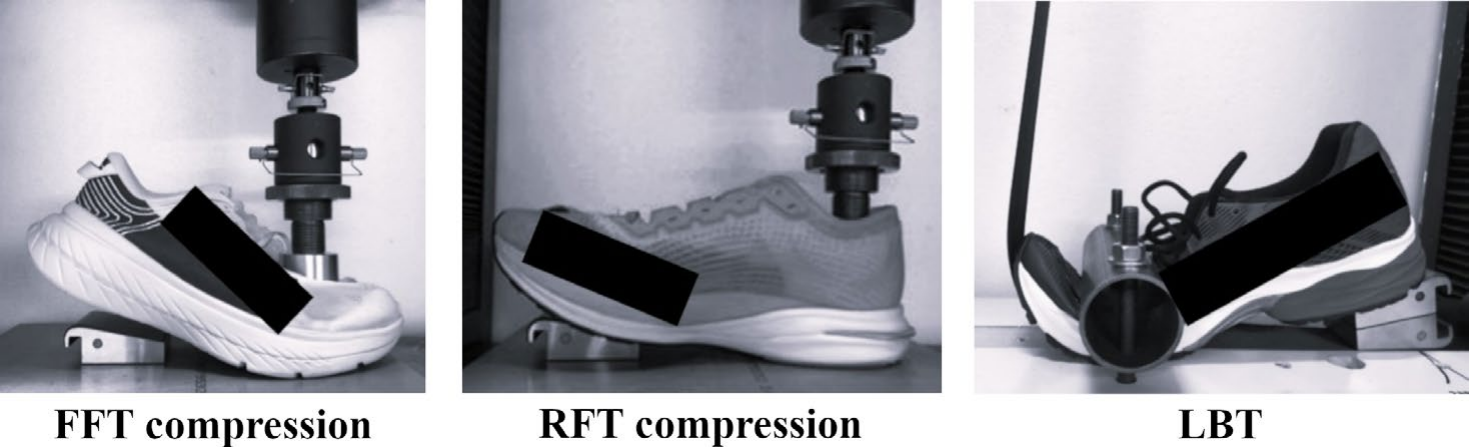
Photographs of the energy return tests (not the same shoes as in the present study): FFT compression—forefoot compression test, RFT compression—rearfoot compression test, LBT—longitudinal bending test. In the compression tests, a load was applied to the shoe at a constant rate of 1 mm·s^−1^, while measuring load and displacement and continued until a maximum load of 1000 N was reached. The load was then reversed at the same rate. In the longitudinal bending test, a belt was fixed behind the shoe, passed beneath the sole, around the toe, and vertically upward to a testing machine. The belt was pre‐tensioned to 2 N and pulled upward at a constant rate of 1 mm·s^−1^ until a displacement of 300 mm was achieved.

Data from two shoe conditions were used in the present study, an AFT (Nike Air Zoom Alphafly Next%) and a non‐AFT (Brooks Levitate 5) shoe (Table 2). The AFT shoe was selected based on energy return (ER) testing (Figure 3) data from 56 different shoe models available on the market in 2022. The chosen AFT shoe demonstrated the highest ER of these shoes. In addition, within the broader testing protocol, this AFT shoe showed the lowest average EC across participants. The non‐AFT shoe was chosen as a reference because its midsole is comprised of standard EVA foam, and it does not contain a longitudinal rigid element.

**TABLE 2:** Shoe properties, measured for size EUR 42.

	Mass [g]	THK RFT	THK FFT	ER_A_ RFT	ER_R_ RFT	ER_A_ FFT	ER_R_ FFT	ER_A_ LBT	ER_R_ LBT
AFT	205	37	35	5.84	83.3	5.29	82.4	3.06	78.3
Non‐AFT	292	32	25	4.55	71.5	2.93	69.1	1.66	64.7

*Note:* THK—thickness (mm), measured at 12% of the shoe's internal length from heel (rearfoot; RFT) and at 75% of the shoe's internal length from heel (forefoot; FFT). ER_A_—absolute energy return (J); ER_R_—relative energy return (%) measured in a compression test at RFT and FFT. LBT—longitudinal bending test, measured at FFT.

All shoe tests, presented in Figure 3, were conducted in three full load cycles from which the absolute energy return (ER_A_) and the relative energy return (ER_R_) were derived only from the third cycle, in each of the tests, in order to reduce initial hysteresis effects. The two compressive tests were conducted at the same positions where the thickness was measured. For the forefoot compression test, the shoe upper was included, since the shoes had to be kept intact. Care was taken, however, to flatten out the shoe upper before the test.

### 2.7 Statistical Analysis

All statistical analyses were performed using R (version 4.3.1; R Foundation for Statistical Computing, Vienna, Austria) in RStudio (version 2023.09.1+494). Means and standard deviations (mean ± SD) were calculated for all variables. EC [W·kg^−1^], ΔEC [%] at three running speeds (60%, 70%, and 80% of VO_2peak_ speed), and all anthropometric parameters were tested for normality using the Shapiro–Wilk test.

Two‐tailed independent samples *t*‐tests were used to compare sex differences for EC and ΔEC. Linear regression analyses were conducted to examine the relationship between each anthropometric characteristic and ΔEC. Additionally, for ΔEC, one‐way repeated measures ANOVA was conducted to determine if the gains from the AFT shoes change with running speed.

Effect sizes were calculated using Cohen's *d*, with values interpreted as small (0.2), medium (0.5), and large (0.8) effects.

## 3 Results

The RER values exceeded 1.0 for one female at 70% and 80% of VO_2peak_ speed, and one male at 80% of VO_2peak_ speed. One female participant's VO_2_ data were excluded due to equipment malfunction.

**FIGURE 4:**
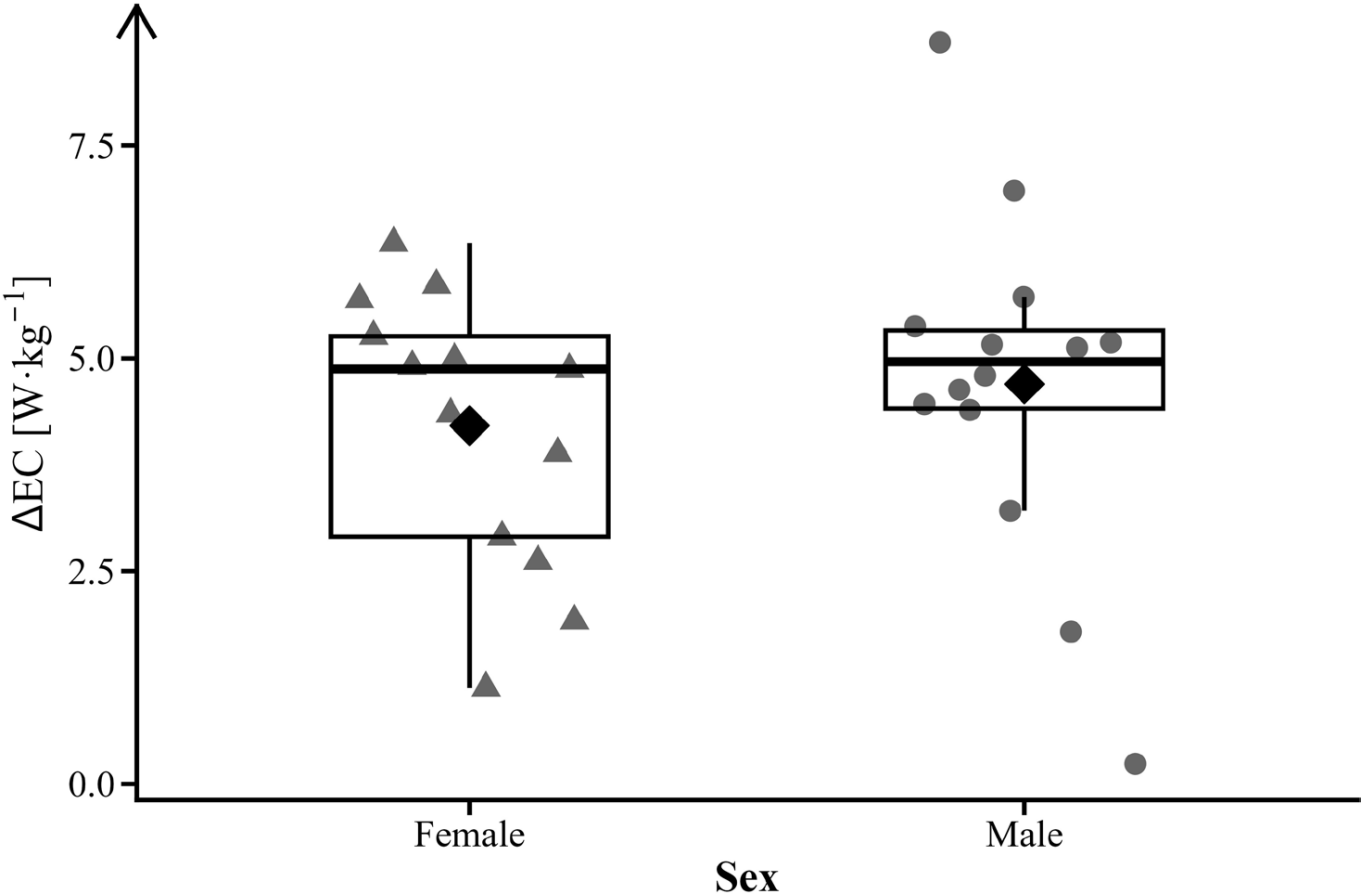
Average change in energy cost (ΔEC) in females (▲) and males (●). Each data point represents the participants' average ΔEC across all three running speeds. Group distributions are shown by median, interquartile range, and mean (◆).

Energetic cost comparisons between sexes showed that females have significantly lower EC values in both shoe conditions, with large effect sizes at all running speeds. However, no significant differences were observed in ΔEC [%] between sexes at any of the tested speeds (Table 3), with small or negligible effect sizes. The average individual ΔEC across all three speeds ranged from 1.13% to 6.35% for females and from 0.24% to 8.71% for males, with group means of 4.21% ± 1.6% for females and 4.70% ± 2.0% for males (*p* = 0.50, Cohen's *d* = 0.26; Figure 4). Furthermore, ΔEC did not vary significantly across speeds for either sex (females: *F*(2, 24) = 1.34, *p* = 0.28; males: *F*(2, 26) = 0.06, *p* = 0.94).

**TABLE 3:** Sex comparison of energetic cost during running in non‐AFT and AFT shoes and the change in energetic cost.

	Speed (%)	Female	Male	*t*	*p*	Cohen's *d*
Non‐AFT (W·kg^−1^)	60	12.92 ± 1.1	14.29 ± 1.2	3.41	0.002*	1.27
	70	15.39 ± 1.1	17.52 ± 1.4	4.41	< 0.001*	1.67
	80	18.11 ± 1.3	20.73 ± 1.9	4.23	< 0.001*	1.63
AFT (W·kg^−1^)	60	12.39 ± 0.9	13.62 ± 1.0	3.52	0.002*	1.31
	70	14.68 ± 1.0	16.68 ± 1.3	4.56	< 0.001*	1.73
	80	17.34 ± 1.2	19.75 ± 1.6	4.34	< 0.001*	1.67
ΔEC (%)	60	4.02 ± 2.4	4.59 ± 2.3	0.64	0.53	0.24
	70	4.56 ± 2.1	4.74 ± 2.3	0.22	0.83	0.08
	80	4.23 ± 1.4	4.63 ± 2.1	0.58	0.57	0.22

*Note:* Data presented as: mean ± SD.

* *p* < 0.05.

Regression analyses (Table 4) showed that none of the anthropometric parameters significantly predicted ΔEC, and the interaction term indicated that the direction of response—whether greater values were associated with higher or lower ΔEC—did not differ between sexes. Corresponding scatter plots illustrating these relationships are presented in Figure S1, which show individual variability and lack of linear associations.

**TABLE 4:** Linear regression analysis of the relationship between anthropometric traits and change in energy cost (ΔEC) by sex.

	Female		Male		Sex interaction	
	*β*	*p*	*β*	*p*	*p*	*R* ^2^
Height	−0.02	0.86	0.03	0.72	0.74	0.025
Weight	−0.06	0.62	0.01	0.91	0.63	0.030
Foot	−0.22	0.77	−0.40	0.26	0.83	0.068
Femur	−0.01	0.98	−0.20	0.38	0.63	0.052
Tibia	−0.02	0.97	0.05	0.84	0.89	0.020
AT	−0.38	0.27	−0.20	0.40	0.67	0.097

*Note:* Weight is reported in kilograms, and all other parameters in centimeters, *β* represents the unstandardized regression coefficients, where positive values indicate increased ΔEC and negative values indicate reduced ΔEC. The “Sex Interaction” column shows whether the association between anthropometric variables and ΔEC differs by sex.

## 4 Discussion

This study investigated sex‐based differences in the change in energy cost (ΔEC) when using an AFT shoe compared to a non‐AFT shoe, and whether these differences were associated with anthropometric characteristics in highly trained long‐distance runners. We hypothesized that (1) females would exhibit a greater ΔEC benefit than males, and (2) specific anthropometric traits would influence ΔEC, with these associations differing between sexes.

Our results did not support the first hypothesis. Although females consistently showed lower absolute EC values than males across all speeds and both shoe conditions, likely due to running at lower absolute speeds, ΔEC did not differ significantly between sexes. ΔEC was 4.21% ± 1.61% for females and 4.70% ± 2.04% for males, with no statistically significant difference between the groups. Previous comparisons between sexes have yielded mixed results. Daniels and Daniels [30] found males to be more economical at absolute intensities, but not at relative intensities, whereas Joubert et al. [31] reported that females were more economical at 10 km·h^−1^, but not at 12 km·h^−1^. These inconsistencies suggest that runner level, testing speed, and physiological factors, such as substrate utilization differences between sexes [32] may influence EC. More recently, with the emergence of AFT shoes, the focus has shifted to improvements in RE or EC across shoe conditions. Barnes and Kilding [3], Joubert et al. [31], and Riedl et al. [10] reported no sex‐based differences in ΔRE at matched absolute or relative intensities, while Matties and Rowley [33] showed a significant difference in ΔEC, but not in ΔRE. Thus, although one study suggested a potential sex difference in energetic cost, the current evidence does not indicate sex‐specific responsiveness to AFT shoes.

It has been suggested that the magnitude of improvement in RE increases with speed, up to around 13 km·h^−1^ [31], after which the benefit tends to plateau [2, 3, 6]. In contrast, Matties and Rowley [33] observed an inverse relationship between RE and running speed, although their participants ran at self‐selected paces. In the present study, the average running speeds ranged from 10.2 to 13.7 km·h^−1^ for females and 12.0 to 16.0 km·h^−1^ for males, corresponding to 60% to 80% of VO_2peak_ speed. Despite this range, we observed no significant differences in ΔEC across speeds for either sex. This is consistent with findings by Hebert‐Losier et al. [5], who also reported no speed‐related differences in ΔEC across a similar speed range (11–14.7 km·h^−1^) when speeds were matched to the same relative intensities. Together, these two studies suggest that the potential relationship between speed and responsiveness in AFT may be more complex and potentially influenced more by relative effort rather than absolute speed. Additionally, differences in shoe models, runner level, and testing protocols across studies may contribute to the variability in reported outcomes.

Several anthropometric traits, such as body mass, shoe size, and leg length, have been proposed as potential contributors to improved performance in AFT shoes, particularly among elite runners [8, 9], and have also been linked to RE [31]. In the present data, all measured anthropometric variables differed significantly between sexes (Table S1). These differences allowed us to explore further how distinct anthropometric profiles might influence ΔEC.

Individual anthropometrics are thought to influence how runners interact with specific shoe components. For example, the midsole foam's energy return might depend on the degree of compression, which is influenced by body mass. Heavier runners might compress the foam more, therefore increasing the energy return, while lighter runners might not reach the optimal deformation range. Conversely, excessive compression could lead to “bottoming out”, where energy return becomes less efficient [31]. Similarly, body mass may affect the optimal stiffness of the rigid plate, as suggested by Roy and Stefanyshyn [34]. However, while these mechanisms are theoretically plausible, the evidence remains inconsistent. Riedl et al. [10] reported a small correlation between body mass and ΔEC, while Van Hooren et al. [25] and Joubert et al. [31] found no such relationship, findings that align with those presented in this study.

Beyond body mass, other anthropometric characteristics, such as foot and leg length, may influence how runners interact with AFT shoe geometry. If AFT shoes are not scaled proportionally in stack height across sizes, then runners with smaller feet may experience relatively larger heel‐to‐toe drop, which, in turn, could alter the timing and location of energy storage and return, potentially affecting ΔEC [2, 35]. Moreover, smaller shoes may also have relatively greater cushioning material, which could increase the amount of energy‐returning foam and potentially enhance energy return, leading to increased ΔEC. Additionally, differences in foot positioning and ankle joint angles during the stance phase may influence muscle activation patterns and joint power, particularly in runners with smaller feet [36]. Matties and Rowley [33] found a negative relationship between shoe size and ΔRE, although their analysis combined runners from both sexes. In contrast, Van Hooren et al. [25] found no relationship between foot length and EC improvement, aligning with our results in both combined (see Table S2) and stratified data. Since Matties and Rowley [33] showed generally lower benefit for males, the observed effect might reflect sex difference, rather than shoe size alone. They hypothesize that a smaller shoe size leads to increased MTP joint power responsiveness. Furthermore, for shorter runners, the same absolute sole thickness may result in a relatively greater increase in height and leg length, potentially leading to an increased step length [8], which has been associated with improved RE in AFT shoes [36]. However, Bertschy et al. [37] reported no difference in EC across shoes with varying stack heights, and similarly, Koegel et al. [38], found no differences in height, body mass, or leg length between runners who improved their RE and those who did not.

Cushioning and longitudinal bending stiffness have also been linked to AT stretch, where softer midsoles may increase ankle dorsiflexion and therefore tendon stretch, potentially improving ΔEC [39]. However, AFT shoes typically reduce peak ankle dorsiflexion [36], which may limit tendon stretch and diminish energy return via this mechanism. Increased longitudinal bending stiffness, a further characteristic of AFT, has, on the other hand, been associated with improved AT energy return [40].

Although longer Achilles tendons, whether expressed as absolute length or relative to leg length, have been associated with better running performance, they also allow for greater energy storage, which in turn reduces relative stretch and results in smaller ankle range of motion [41].

Although longer Achilles tendons, whether expressed as absolute length or relative to leg length, are linked to better running performance due to greater energy storage [42], they also result in reduced relative stretch and smaller ankle range of motion [41]. Since AFT shoes also tend to reduce ankle dorsiflexion, they may replicate the effects of long AT. This overlap could theoretically mean that runners with longer tendons experience smaller ΔEC benefits from AFT shoes, as the shoe compensates for their natural advantage. However, data from this study showed no significant relationship between AT length and ΔEC.

The results of this study, contrary to the second hypothesis, suggest that commonly proposed anthropometric characteristics do not significantly predict the energetic benefit of AFT shoes in highly trained runners, aligning with inconsistent or weak associations [25, 31]. While theoretical models suggest that individual morphology may influence how runners interact with shoe components, our results indicate that these effects might be more nuanced or overshadowed by other factors. Moreover, the lack of significant findings may reflect the relatively homogeneous nature of our sample—highly trained distance runners with limited variation in performance and anthropometric range. These observations highlight the complexity of the runner‐shoe interaction and suggest that a single characteristic, either of the runner or the AFT shoe, in isolation is unlikely to explain individual responsiveness to AFT footwear.

This study has several limitations that should be considered when interpreting the findings. First, the AFT and non‐AFT shoes used in this study were not mass‐matched, and shoe mass was not accounted for in the ΔEC calculations, which may have slightly inflated the results [23] compared to studies where shoe mass was matched. This decision was made to preserve ecological validity, as the shoes were tested in their commercially available form. Second, although the inter‐individual variability observed was smaller than previously reported, it should still be interpreted with caution. Limited familiarization with the shoes, combined with a warm‐up performed in participants' personal shoes, may have contributed to this variability [43], and prior research suggests that at least two trials per shoe are needed to reliably assess RE in AFT shoes [44], however, this was not feasible given the four shoe conditions and the requirement for 12‐min runs in each. Additionally, running at intensities likely exceeding the first lactate or ventilatory threshold may induce a VO_2_ slow component. Although lactate was measured, individual thresholds were not determined. Despite randomization of shoe order, time‐dependent physiological drift may have increased within‐subject VO_2_ variability, reducing sensitivity to detect small differences between shoes. While the relatively homogeneous sample of runners permits conclusions relevant to this specific population, it limits the generalization, potentially reducing the likelihood of detecting meaningful associations. Additionally, while treadmill testing offers controlled conditions, it may not fully replicate overground running, as treadmills mostly have higher shock absorption and smaller energy return [45]. Finally, although females have recently shown greater reductions in race times, which has been closely linked to AFT footwear, this was not reflected in EC improvements in this study. This disconnect may suggest that factors beyond EC, such as advancements in training strategies or broader sociocultural shifts, also contribute to performance improvements. Future research should explore broader populations and investigate whether combined models can better predict individual responsiveness to AFT footwear.

In conclusion, this study found no significant associations between anthropometric characteristics and the change in energetic cost in AFT shoes in highly trained long‐distance runners. While females demonstrated lower absolute EC values than males, the relative improvements in EC showed no significant difference across sexes and running speeds. These findings suggest that individual responsiveness to AFT footwear cannot be explained by the isolated anthropometric traits explored in this study.

## 5 Perspective

The present findings contribute to the growing body of research on AFT by exploring two pathways for individual responsiveness: sex and anthropometric characteristics. While previous studies have reported consistent average improvements in running economy with AFT shoes, individual inter‐variability remains largely unexplored. Results from this study indicate that neither sex nor isolated anthropometric characteristics could explain differences in ΔEC among highly trained runners, suggesting that individualization of AFT footwear should not be based on sex or the investigated anthropometric characteristics. Future research should focus on other factors, for example individual running technique and neuromuscular function. These findings highlight the complexity of AFT individualization. Ultimately, understanding the connection between shoe design and individual responsiveness could greatly improve individual athletic performance.

## Funding

This work was supported by The Swedish Research Council (Grant 2021‐04437) and The Swedish Research Council for Sport Science (Grant P2025‐0091).

## Conflicts of Interest

The authors declare no conflicts of interest.

## Supporting information


**FIGURE S1:** Relationship between change in energy cost (ΔEC) and anthropometric characteristics for females (▲) and males (●), Dashed and dotted lines represent sex‐specific linear regression fits for females and males, respectively, illustrating that the direction of response did not differ between sexes.

**TABLE S1:** Anthropometric comparison between females and males. Data presented as: mean ± SD; *p < 0.05.

**TABLE S2:** Linear regression analysis of the relationship between anthropometric traits and change in energy cost (ΔEC). Weight is reported in kilograms, and all other parameters in centimeters, *β* represents the unstandardized regression coefficients, where positive values indicate increased ΔEC and negative values indicate reduced ΔEC.

## Data Availability

The data that support the findings of this study are available from the corresponding author upon reasonable request.
