# Supplementary figures and images for: Sex and Isolated Anthropometric Measures Do Not Explain Individual Differences in Responsiveness to Advanced Footwear Technology in Highly Trained Runners
Source: Scand J Med Sci Sports. 2026 Feb 22;36(2):e70234. doi: 10.1111/sms.70234 (PMC12926519; doi:10.1111/sms.70234)

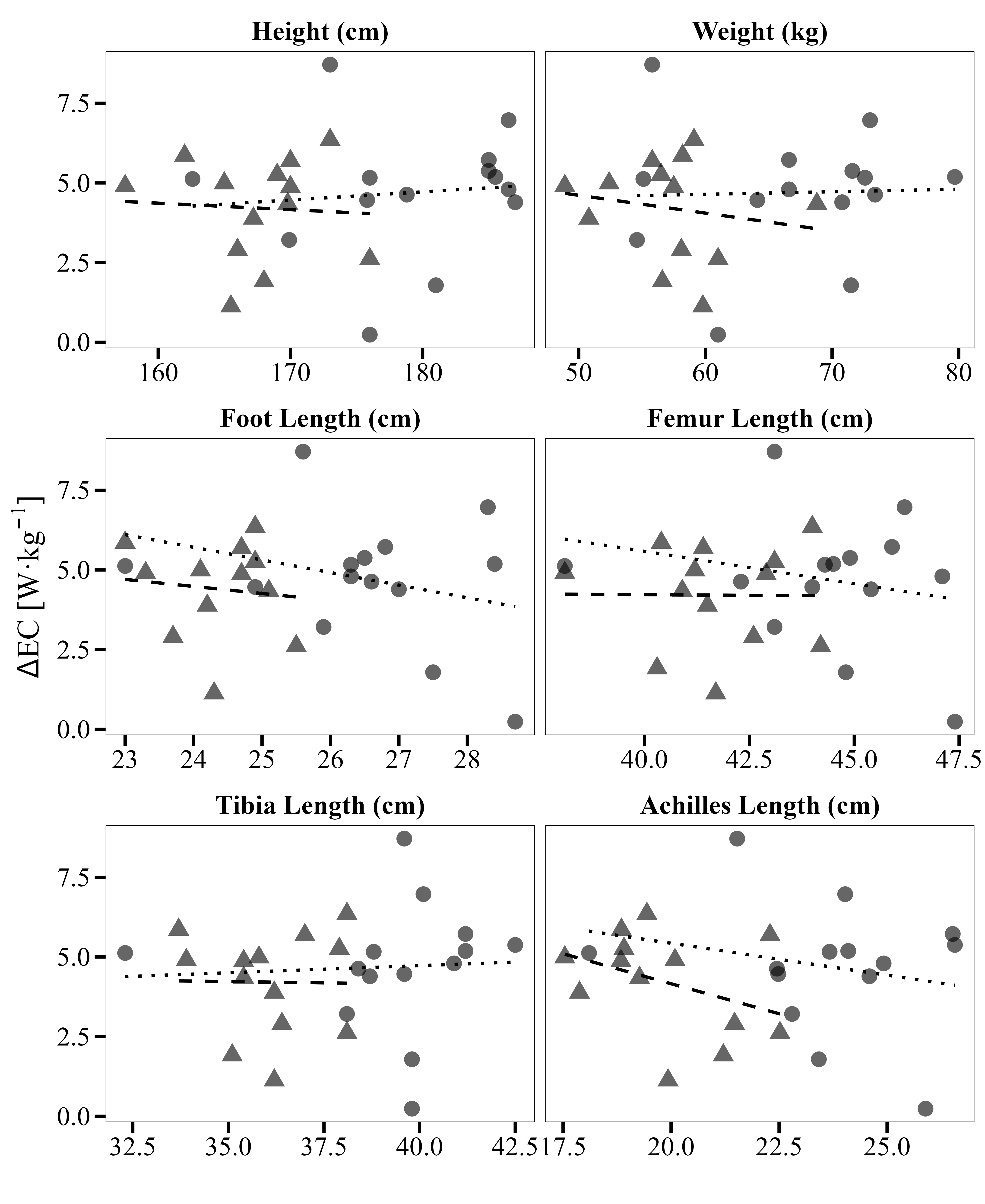

Supplement: Supplementary file 1 — FIGURE S1: Relationship between change in energy cost (ΔEC) and anthropometric characteristics for females (▲) and males (●), Dashed and dotted lines represent sex‐specific linear regression fits for females and males, respectively, illustrating that the direction of response did not differ between sexes. [file SMS-36-e70234-s002.tiff]
