# Supplementary material for: Sex and Isolated Anthropometric Measures Do Not Explain Individual Differences in Responsiveness to Advanced Footwear Technology in Highly Trained Runners
Source: Scand J Med Sci Sports. 2026 Feb 22;36(2):e70234. doi: 10.1111/sms.70234 (PMC12926519; doi:10.1111/sms.70234)
Supplement: Supplementary file 2 — TABLE S1: Anthropometric comparison between females and males. Data presented as: mean ± SD; *p < 0.05. [file SMS-36-e70234-s001.docx]

Supplementary Table S1. Anthropometric comparison between females and males. Data presented as: mean ± SD; * p < 0.05

|  | **Female** | **Male** | **t** | **p** | **Cohen’s d** |
| --- | --- | --- | --- | --- | --- |
| Height (cm) | 167.6 ± 4.70 | 179.37 ± 7.16 | 4.89 | <.001* | 1.85 |
| Weight (kg) | 56.98 ± 4.85 | 67.15 ± 7.58 | 4.27 | <.001* | 1.59 |
| Foot (cm) | 24.30 ± 0.76 | 26.56 ± 1.44 | 5.07 | <.001* | 1.92 |
| Femur (cm) | 41.70 ± 1.60 | 44.49 ± 2.30 | 3.76 | .001* | 1.40 |
| Tibia (cm) | 36.04 ± 1.40 | 39.32 ± 2.29 | 4.70 | <.001* | 1.72 |
| AT (cm) | 20.07 ± 1.71 | 23.46 ± 2.25 | 4.55 | <.001* | 1.69 |

Note: Independent samples t-tests were used to compare values between sexes after confirming normality.
