# Supplementary material for: Sex and Isolated Anthropometric Measures Do Not Explain Individual Differences in Responsiveness to Advanced Footwear Technology in Highly Trained Runners
Source: Scand J Med Sci Sports. 2026 Feb 22;36(2):e70234. doi: 10.1111/sms.70234 (PMC12926519; doi:10.1111/sms.70234)
Supplement: Supplementary file 3 — TABLE S2: Linear regression analysis of the relationship between anthropometric traits and change in energy cost (ΔEC). Weight is reported in kilograms, and all other parameters in centimeters, β represents the unstandardized regression coefficients, where positive values indicate increased ΔEC and negative values indicate reduced ΔEC. [file SMS-36-e70234-s003.docx]

Supplementary Table S2. Linear regression analysis of the relationship between anthropometric traits and change in energy cost (ΔEC). Weight is reported in kilograms, and all other parameters in centimeters, β represents the unstandardized regression coefficients, where positive values indicate increased ΔEC and negative values indicate reduced ΔEC.

|  | **Pooled population** | | |
| --- | --- | --- | --- |
|  | **β** | **p** | **R^2^** |
| Height | 0.02 | 0.65 | 0.009 |
| Weight | 0.01 | 0.78 | 0.003 |
| Foot | -0.13 | 0.56 | 0.014 |
| Femur | -0.04 | 0.80 | 0.003 |
| Tibia | 0.08 | 0.58 | 0.013 |
| AT | -0.06 | 0.65 | 0.008 |
